# Supplementary material for: Morphoanatomical Changes in Eucalyptus grandis Leaves Associated with Resistance to Austropuccinia psidii in Plants of Two Ages
Source: Plants (Basel). 2023 Jan 12;12(2):353. doi: 10.3390/plants12020353 (PMC9867522; doi:10.3390/plants12020353)
Supplement: Supplementary file 1 [file plants-12-00353-s001.zip › plants-2022813-supplementary.pdf]

1. Methodology of collecting leaf samples in the field (Figure S1 above)

Eucalyptus branches were collected on 6 and 20 months old plantats and are represented (Figure S1 above). Leaves of these branches were numbered according to their positions on the branch and detached and separated in five identified plastic boxes, with the same numbers as the leaf position on the branches where they were collected.

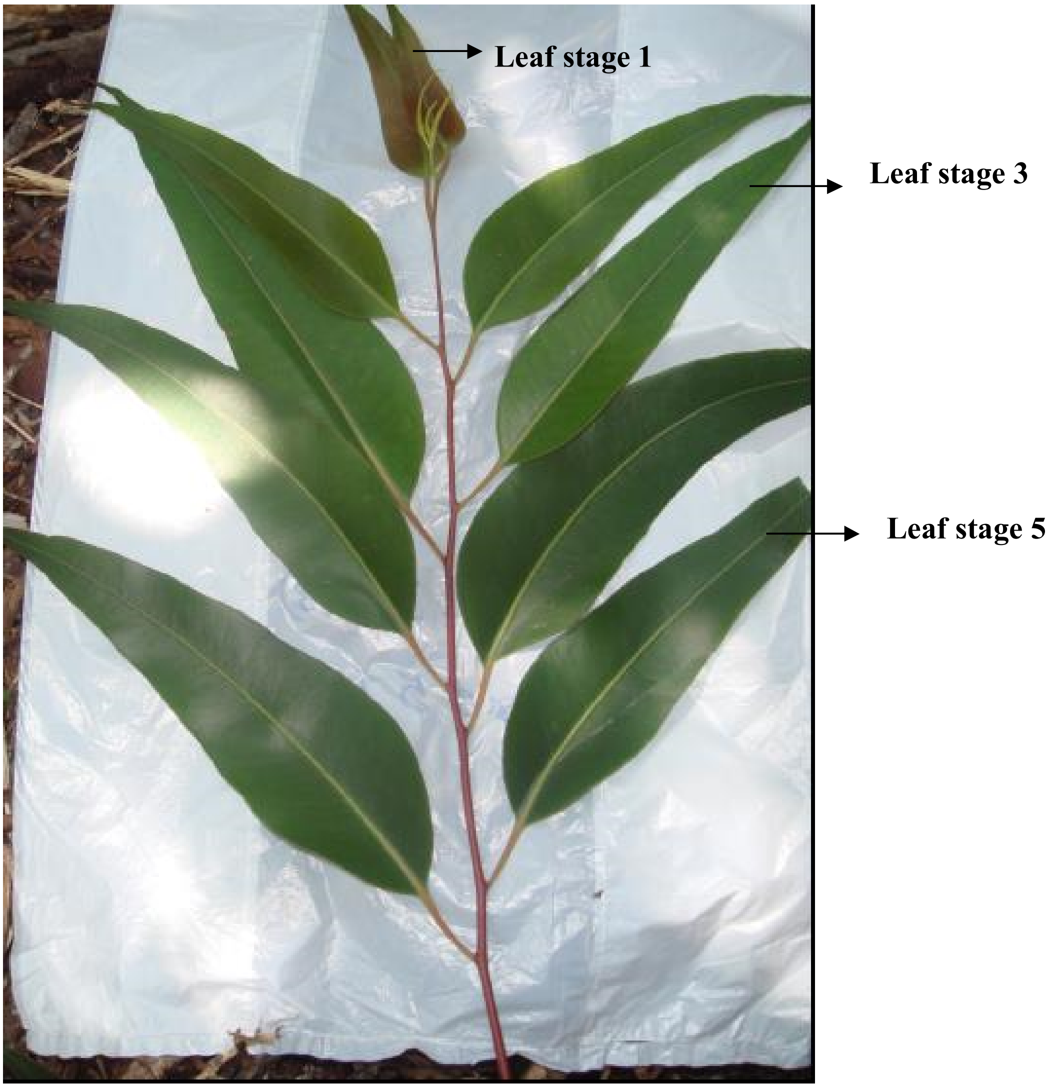

Figure S1. Eucalypt branch with leaves of different development stages collected on 6 and 20 months old plants.

2. Classifying leaf samples by comparing them through color and dimensions (length or width) or with graphic interpolation

The representative visual scale obtained (Figure 1 of the article) makes possible to classify eucalypts leaves in terms of its development stage by comparing them through color and dimensions (length or width) or by graphic interpolation (Table S1 and Figure S2 above).

Table S1. Length (cm) and width (cm) of eucalytp leaves at five development stages

Development stages	Length (cm)	Width (cm)
**Leaf 1**	1,4	0,4
**Leaf 2**	4,1	1,2
**Leaf 3**	5,7	2,6
**Leaf 4**	8,7	3,6
**Leaf 5**	10,2	5

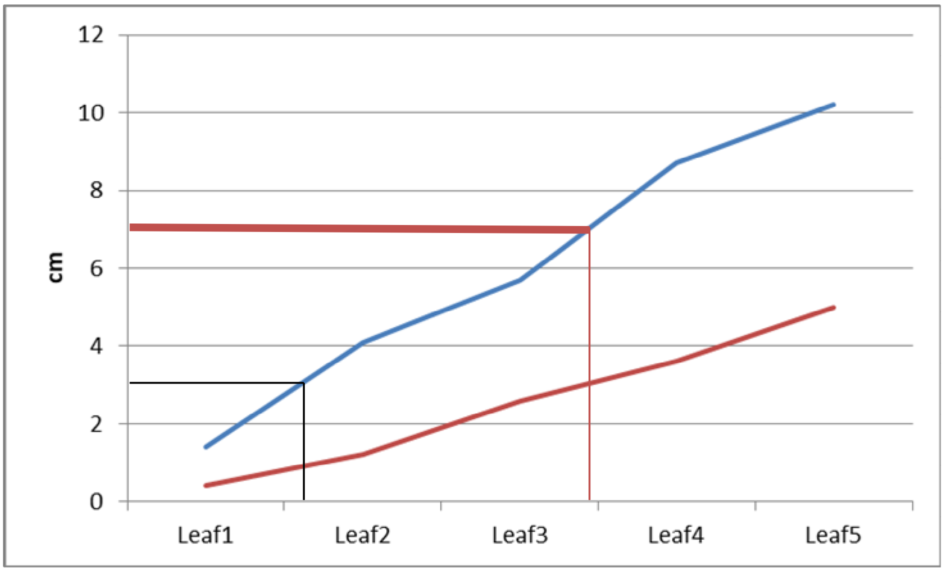

Figure S2. Length (blue) and width (red) of eucalyptus leaves at five development stages.

For example: the identification of 3 cm long leaf is done graphically by plotting this value on the ordinate axis and obtaining leaf 2 on the abscissa axis (Figure S2). The same procedure is used for another leaf with 7 cm long close to the transition between leaf 3 to 4 and soon on.

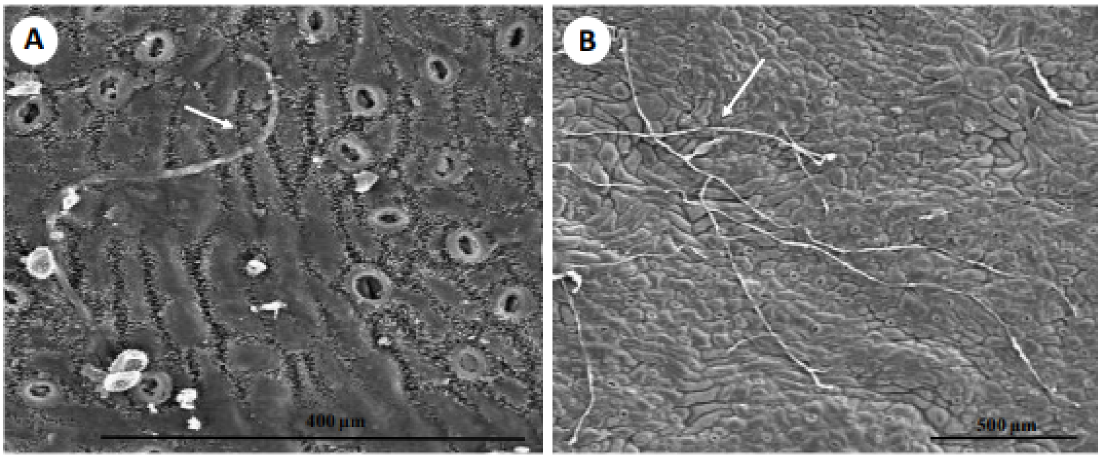

Figure S3. Abaxial surface of a Eucalyptus grandis clone 120 leaf in the fifth development stage after the inoculation of Austropuccinia psidii. Resistant clone with shriveled spores (arrow) without appressorium or pathogen penetration (arrow) (A). Susceptible clone with extensive germ tube formation without appressorium or pathogen penetration (arrow) (B). (SilvaSouza, et al., 2017, doi:10.3390/f8100362).
